# LEDGF_1-326_ Decreases P23H and Wild Type Rhodopsin Aggregates and P23H Rhodopsin Mediated Cell Damage in Human Retinal Pigment Epithelial Cells

**DOI:** 10.1371/journal.pone.0024616

**Published:** 2011-09-07

**Authors:** Rinku Baid, Robert I. Scheinman, Toshimichi Shinohara, Dhirendra P. Singh, Uday B. Kompella

**Affiliations:** 1 Department of Pharmaceutical Sciences, University of Colorado Anschutz Medical Campus, Aurora, Colorado, United States of America; 2 Department of Ophthalmology, University of Nebraska Medical Center, Omaha, Nebraska, United States of America; 3 Department of Ophthalmology, University of Colorado Anschutz Medical Campus, Aurora, Colorado, United States of America; Johns Hopkins School of Medicine, United States of America

## Abstract

**Background:**

P23H rhodopsin, a mutant rhodopsin, is known to aggregate and cause retinal degeneration. However, its effects on retinal pigment epithelial (RPE) cells are unknown. The purpose of this study was to determine the effect of P23H rhodopsin in RPE cells and further assess whether LEDGF_1-326_, a protein devoid of heat shock elements of LEDGF, a cell survival factor, reduces P23H rhodopsin aggregates and any associated cellular damage.

**Methods:**

ARPE-19 cells were transiently transfected/cotransfected with pLEDGF_1-326_ and/or pWT-Rho (wild type)/pP23H-Rho. Rhodopsin mediated cellular damage and rescue by LEDGF_1-326_ was assessed using cell viability, cell proliferation, and confocal microscopy assays. Rhodopsin monomers, oligomers, and their reduction in the presence of LEDGF_1-326_ were quantified by western blot analysis. P23H rhodopsin mRNA levels in the presence and absence of LEDGF_1-326_ was determined by real time quantitative PCR.

**Principal Findings:**

P23H rhodopsin reduced RPE cell viability and cell proliferation in a dose dependent manner, and disrupted the nuclear material. LEDGF_1-326_ did not alter P23H rhodopsin mRNA levels, reduced its oligomers, and significantly increased RPE cell viability as well as proliferation, while reducing nuclear damage. WT rhodopsin formed oligomers, although to a smaller extent than P23H rhodopsin. Further, LEDGF_1-326_ decreased WT rhodopsin aggregates.

**Conclusions:**

P23H rhodopsin as well as WT rhodopsin form aggregates in RPE cells and LEDGF_1-326_ decreases these aggregates. Further, LEDGF_1-326_ reduces the RPE cell damage caused by P23H rhodopsin. LEDGF_1-326_ might be useful in treating cellular damage associated with protein aggregation diseases such as retinitis pigmentosa.

## Introduction

Intracellular protein aggregation has been linked to many degenerative diseases including retinitis pigmentosa (RP) [1], [2]. Rhodopsin, a protein present in retinal cells is one such protein, which forms aggregates upon cellular accumulation. Some mutations of rhodopsin such as the point mutation P23H (Proline 23 → Histidine) result in greater aggregation [3], [4]. These aggregates cause progressive degeneration of retinal cells, leading to blindness in RP. The P23H point mutation constitutes one of the most common causes of autosomal dominant RP in North America. P23H rhodopsin forms protein aggregates and accumulates as aggresomes in the cytosol, leading to the death of human embryonic kidney cells [3], [5]. P23H rhodopsin also exerts a dominant negative effect on the biosynthesis of normal wild type (WT) rhodopsin and induces formation of WT/P23H rhodopsin aggregates as well P23H/P23H rhodopsin aggregates [6], [7], [8]. While numerous studies have investigated P23H rhodopsin aggregation including its localization, morphology, as well as its effect on the expression of WT rhodopsin [3], [4], [9], [10], there is a distinct lack of methods for potentially reducing the pathological effects of this mutation that may be applied to the clinical setting.

In this study we investigated the ability of LEDGF_1-326_, a fragment of lens epithelium derived growth factor (LEDGF), to prevent cellular damage mediated by P23H rhodopsin and WT rhodopsin. LEDGF, a 530 amino acid (aa) protein originally isolated from a cDNA library of lens epithelial cells [11], is a transcription factor that confers cellular resistance to oxidative and thermal stresses and increases cell survival. Once in the nucleus, it binds to stress response elements and/or heat shock elements present in the promoters of stress associated genes, and thereby upregulates their transcription and expression [12], [13], [14], [15]. LEDGF was also isolated in a screen for proteins that interact with the HIV integrase [16] and appears to be essential for nuclear targeting of the integrase in human cells [17].

**Figure 1:**
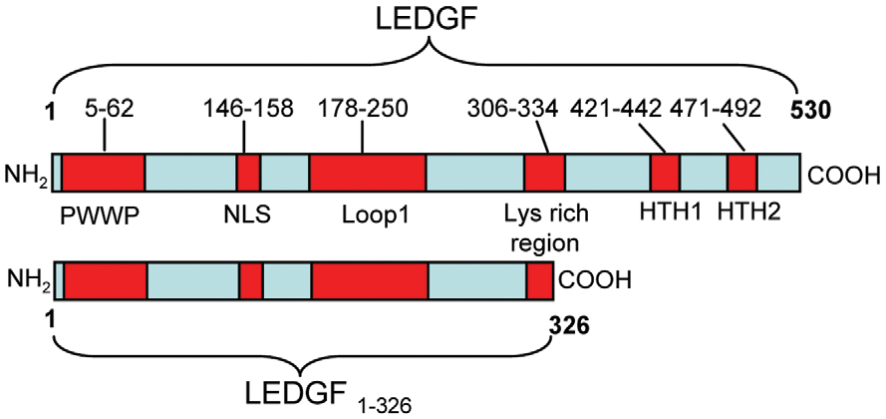
Schematic representation of LEDGF domains. PWWP, DNA binding domain (aa5–62); Nuclear localization signal (aa148–156); Loop1, contains AT hook like domain (aa178–250), lysine rich coiled coil domain (aa216–343), HTH1, Helix-turn-helix domain (aa421–442); HTH2, Helix-turn-helix domain (aa471–492).

Bioinformatic analysis of the structural organization of LEDGF predicted various domains of LEDGF [18]. LEDGF contains an N-terminal DNA binding domain spanning from amino acid aa5 to aa62 (Figure 1). This amino acid region contains a proline-tryptophan-tyrptophan-proline (PWWP) motif that has been shown to bind to stress response elements (STRE; A/TGGGGA/T; A = Alanine, T = Threonine, G =  Glycine) of DNA and transactivate stress related genes. A nuclear localization signal spans aa148 to aa156 (GRKRKAEKQ; R = Arginine, K =  Lysine, E = Glutamic acid, Q = Glutamine). An AT hook-like domain at aa178 to aa197, a looped structure at aa178to aa250, and a coiled coil domain rich in lysine from aa216 to aa343, are also present in LEDGF. The C- terminus of LEDGF contains two helix-turn-helix (HTH) domains at aa421 to aa442 and aa471 to a492 that are capable of binding to heat shock elements (HSE) within the promoters of various genes. The minimum requirement for LEDGF activity appears to be a nuclear binding domain to upregulate various stress related proteins, a nuclear localization sequence to translocate LEDGF fragment to the nucleus, and a stretch of lysine rich residues to assist binding to the DNA [18]. While heat shock proteins are known to act like chaperones in reducing protein aggregation [19], [20], the ability of LEDGF or its derivatives in reducing protein aggregation are unknown. Considering all the requirements for LEDGF activity, we designed LEDGF_1-326_, a fragment of LEDGF, which contained all the functionally important residues of LEDGF, minus the heat shock elements and tested its ability to reduce rhodopsin aggregates and cell damage.

Retinal pigmented epithelial (RPE) cells play an important role in the generation and maintenance of photoreceptors [21]. They are located between the choroid and the photoreceptors of the neural retina. They supply nutrients to the neural retina and phagocytose dead photoreceptors. They also maintain the visual function of photoreceptors. With aging, RPE cells in human eyes are known to accumulate rod outer segments containing rhodopsin, due to a deficiency of rhodopsin degrading enzymes including alpha-mannosidase [22]. Given the propensity of rhodopsin to aggregate, it is possible that rhodopsin accumulation contributes to RPE loss. Indeed, mice expressing rhodopsin mutations such as P23H lose RPE at a much accelerated rate [23]. Since RPE degeneration is an integral part of RP, we chose in this study to more directly examine the effects of rhodopsin aggregates (both P23H and WT) on RPE survival. Another objective of this study was to assess whether LEDGF_1-326_ reduces rhodopsin aggregates as well as the cellular damage induced by rhodopsin proteins in RPE cells leading to decreased survival. Towards these objectives, we performed in vitro transient transfections of P23H/WT rhodopsin and LEDGF_1-326_ plasmids in a human retinal pigmented epithelial cell line (ARPE-19). Subsequently, we performed trypan-blue assay for cell viability, BrDu assay for cell proliferation, confocal microscopy to visualize the proteins and their localization, western blot analysis to quantify rhodopsin monomers and aggregates, and real time quantitative PCR to measure the mRNA level of P23H rhodopsin in the presence and absence of LEDGF_1-326_.

Our work has shown that both P23H and WT rhodopsin form aggregates in retinal pigment epithelial cells and LEDGF_1-326_ is capable of reducing these protein aggregates. Further, LEDGF1-326 decreases P23H rhodopsin mediated cell damage in RPE cells.

## Results

### P23H Rhodopsin decreases RPE cell viability and proliferation

**Figure 2:**
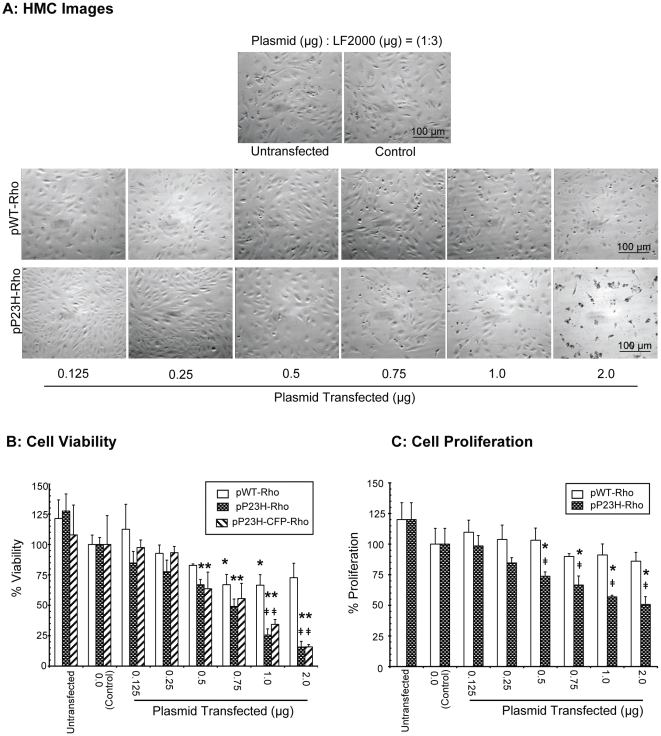
P23H rhodopsin is toxic to RPE cells. **A**) For HMC images, cells were visualized using Nikon inverted light microscope at 10x magnification. Representative images from one of the three independent studies have been shown. **B**) For cell viability, the cells were trypsinized, collected and resuspended in PBS. Total number of viable cells was determined in each group using trypan blue dye and percent viability was calculated with respect to the group transfected with LF2000 (Lipofectamine® 2000) alone. **C**) For cell proliferation cells were treated with BrDU for 24 h and then detected using anti-BrDU antibody. The percentage proliferation was calculated with respect to LF2000 transfected group. Data is expressed as mean ± S.D. for N = 3. *, p< 0.01 compared with corresponding pWT-Rho transfected group. *, p<0.05 compared to LF2000 group.

To determine the effect of P23H rhodopsin expression in ARPE-19 cells, a trypan-blue cell viability assay was done (Figure 2B). Before cells were trypsinized, Hoffman Modulation Contrast (HMC) images were taken of the live cells, using phase contrast microscopy (Figure 2A). The untransfected and LF2000 (Lipofectamine® 2000) transfected (control) groups showed confluent cells. As the transfection level of pP23H-Rho increased, the number of cells per frame of the image decreased.

In our cell viability assay (Figure 2B), cells transfected with LF2000 alone were indistinguishable from cells transfected with, low levels (<0.25 µg) of pP23H-Rho. As the level of transfected pP23H-Rho was increased, we observed a significant decrease in the percentage of viable cells (p<0.001). At transfection level of 1.0 µg, the cell viability was 25±5%, whereas at 2.0 µg, the cell viability was only 15±1%, indicating a significant loss in cell viability (p<0.001). A similar trend was seen when cells were transfected with pP23H-CFP-Rho (P23H rhodopsin tagged with cyan fluorescent protein). Transfection of 1.0 µg of P23H resulted in a cell viability of 34±4%, whereas at 2.0 µg, the cell viability was only 15±2%, indicating a significant loss in cell viability (p≤0.001).

Similar to P23H rhodopsin, as the transfection level of pWT-Rho increased, the number of cells per frame of the image decreased (Figure 2A), but the change was not as prominent as pP23H-Rho transfected groups. While there was no significant difference at 0.25, 0.5, and 0.75 µg doses, there was a significant difference between the cell viability of pWT-Rho and pP23H-Rho/pP23H-CFP-Rho groups at 1 and 2 µg doses. At the 2 µg dose for instance, the viability with pWT-Rho was 72±11% as opposed to 15±1% with pP23H-CFP.

To further understand whether the loss of viability of cells is due to a decrease in cell proliferation, a BrDU assay was performed (Figure 2C). pP23H-Rho decreased proliferation of ARPE-19 cells in a dose-dependent manner when compared to LF2000 controls, with the % proliferation being 50±6% at 2 µg, the highest dose tested. Although there appeared to be a decline in cell proliferation with pWT-Rho, the differences were not statistically significant. Cells transfected with pP23H-Rho showed significantly lower proliferation when compared to cells transfected with pWT-Rho at all doses. Although there was no dose dependent decline in cell viability with pWT-Rho, the decline in viability was significant with transfections of 0.75 and 1 µg DNA, when compared to controls.

### LEDGF_1-326_ decreases RPE cell viability loss induced by P23H rhodopsin

**Figure 3:**
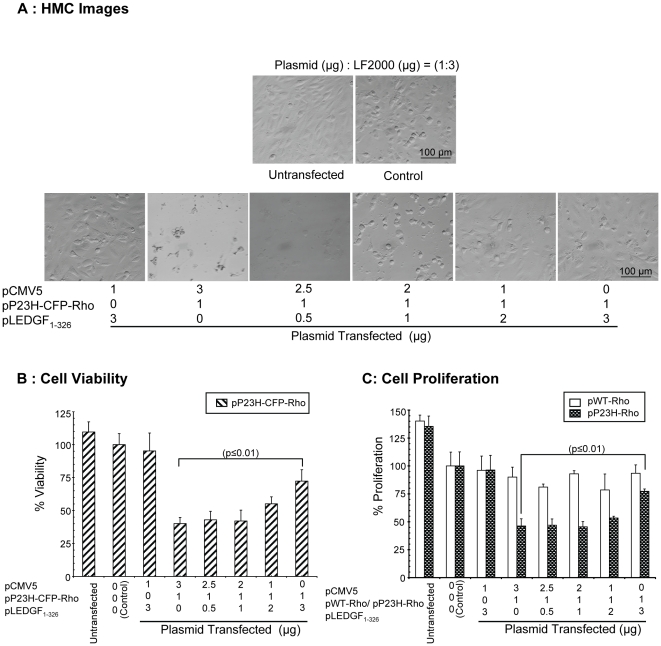
LEDGF_1-326_ increases the cell viability and proliferation in a dose-dependent manner in RPE cells expressing P23H rhodopsin. **A**) For HMC images, cells were visualized using Nikon inverted light microscope at 10x magnification. Representative images from one of the three independent studies have been shown. **B**) For cell viability, cells were trypsinized, collected and resuspended in PBS. Total number of viable cells was determined in each group using trypan blue assay and percent viability was calculated with respect to the group transfected with LF2000 (Lipofectamine® 2000) alone. **C**) For cell proliferation assay, cells were treated with BrDU for 24 h and then detected using anti-BrDU antibody. The percentage proliferation was calculated with respect to LF2000 transfected group. Data is expressed as mean ± S.D. for N = 3. p<0.01 compared with LF2000 transfected (control) group.

To demonstrate the ability of LEDGF_1-326_ to rescue cells from rhodopsin induced damage, ARPE-19 cells were cotransfected with a constant dose of pP23H-CFP-Rho/pP23H-Rho/pWT-Rho and increasing doses of pLEDGF_1-326_. An empty plasmid (pCMV5) was introduced to keep the total transfected plasmid constant in the various treatment groups. Trypan-blue cell viability assays (Figure 3B) and BrDU cell proliferation assays (Figure 3C) were then performed. Further, HMC images (Figure 3A) were taken in those groups with fluorescently tagged proteins. Figure 3A represents images from one of the three independent studies. The HMC images indicated a significant loss in number of cells in groups transfected with pP23H-CFP-Rho alone. As the cotransfection level of pLEDGF_1-326_ was increased, cell loss commensurably decreased.

The percentage viability of ARPE-19 cells in the group transfected with pP23H-CFP-Rho alone was 40±5% as compared to the LF2000 transfected group (Figure 3B). In the presence of pLEDGF_1-326_, cell viability increased in a dose dependant manner. As the level of cotransfected pLEDGF_1-326_ was increased from 0 to 3 µg, the percentage cell viability increased from 40±5 to 72±8%, that is, about ∼1.7 times (p<0.01). However, cell viability could not be restored to 100% even at the highest dose of pLEDGF_1-326_.

In the BrDU cell proliferation assay (Figure 3C), cell proliferation in the group transfected with pP23H-Rho was low (46±6%) as compared to the LF2000 transfected group. When pLEDGF_1-326_ was cotransfected with pP23H-Rho, there was an increase in cell proliferation in a dose dependent manner. The % proliferation of pP23H-Rho transfected cells increased from 46±6 to 77±2% at the highest dose of pLEDGF_1-326_. On the other hand, pWT-Rho transfected groups did not show any significant changes in the proliferation in the absence or presence of pLEDGF_1-326_.

### P23H rhodopsin disrupts RPE nuclear shape and content

**Figure 4:**
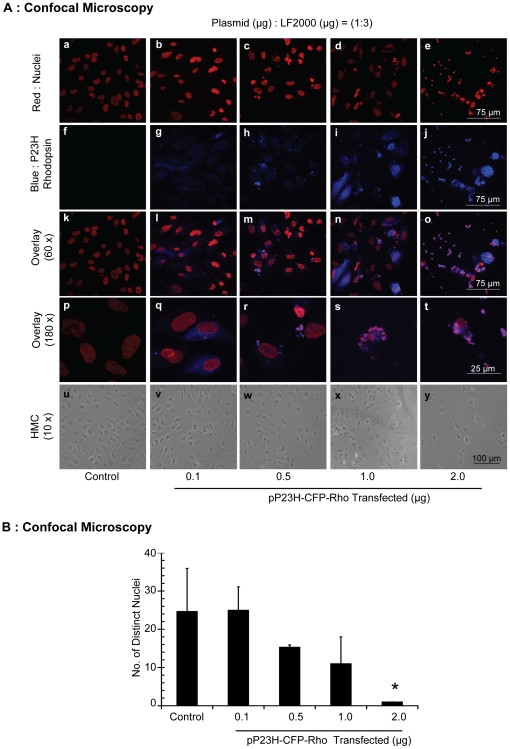
P23H rhodopsin disrupts the nuclear material and aggregates in RPE cells. **A**) For confocal images, cells were fixed with 4% buffered formalin, permeabilized with 0.2% Triton X-100, and treated with RNase. The nucleus was then stained with TO-PRO-3 iodide. Confocal images were taken using the excitation/emission wavelengths of 637–605/75 and 408–450/35 nm for TO-PRO-3 iodide and CFP, respectively. Representative images from one of the three independent studies have been shown. For HMC images, the cells were visualized using Nikon inverted light microscope at 10x magnification. **B**) The number of distinct nuclei was counted in each confocal image from three independent studies and was plotted against the pP23H-CFP-Rho transfection level. Data is expressed as mean ± S.D. for N = 3. *, p<0.01 compared with LF2000 transfected (control) group.

We assessed the effect of P23H rhodopsin expression on ARPE-19 cells histologically by confocal microscopy. ARPE-19 cells were cultured on cover slips and transiently transfected with increasing level of pP23H-CFP-Rho. To visualize the nucleus, cells were stained with red TOPRO-3 iodide dye, while P23H rhodopsin expression was represented by blue fluorescence emitted by P23H rhodopsin tagged with CFP (Figure 4A). Representative images from one of the three independent studies are shown. As the level of pP23H-CFP-Rho was increased there was an increase in blue fluorescence. When <0.5 µg of pP23H-CFP-Rho was used for transfection, the nucleus was distinct and well formed (Figure 4A (a, and b)). This distinct nuclear structure (defined as well formed ovals) started disappearing as the transfection level of pP23H-CFP-Rho was increased (Figure 4A (c, d, and e)). When cells were visualized at higher magnification (Figure 4A, Row 4), disrupted nuclear material was observed (Figure 4A (s, and t)). The LF2000 treated control group (Figure 4A, p), in contrast, did not show any such structural damage. HMC images were taken at 10x magnification (Figure 4A, Row 5). As the transfection level of pP23H-CFP-Rho was increased, the number of cells present in a given area decreased, indicating loss of cells. This result correlates both with our cell viability and proliferation data (Figure 2).

Further, to understand the significance of the confocal data, the numbers of distinct nuclei visible in each confocal image were counted from images from three independent studies (Figure 4B). The number of distinct nuclei were significantly reduced in groups transfected with 2.0 µg of pP23H-CFP-Rho as compared to LF2000 transfected control group (p<0.01). Thus, P23H rhodopsin expression disrupts nuclear structure.

### LEDGF_1-326_ rescues RPE cells from nuclear damage induced by P23H rhodopsin

**Figure 5:**
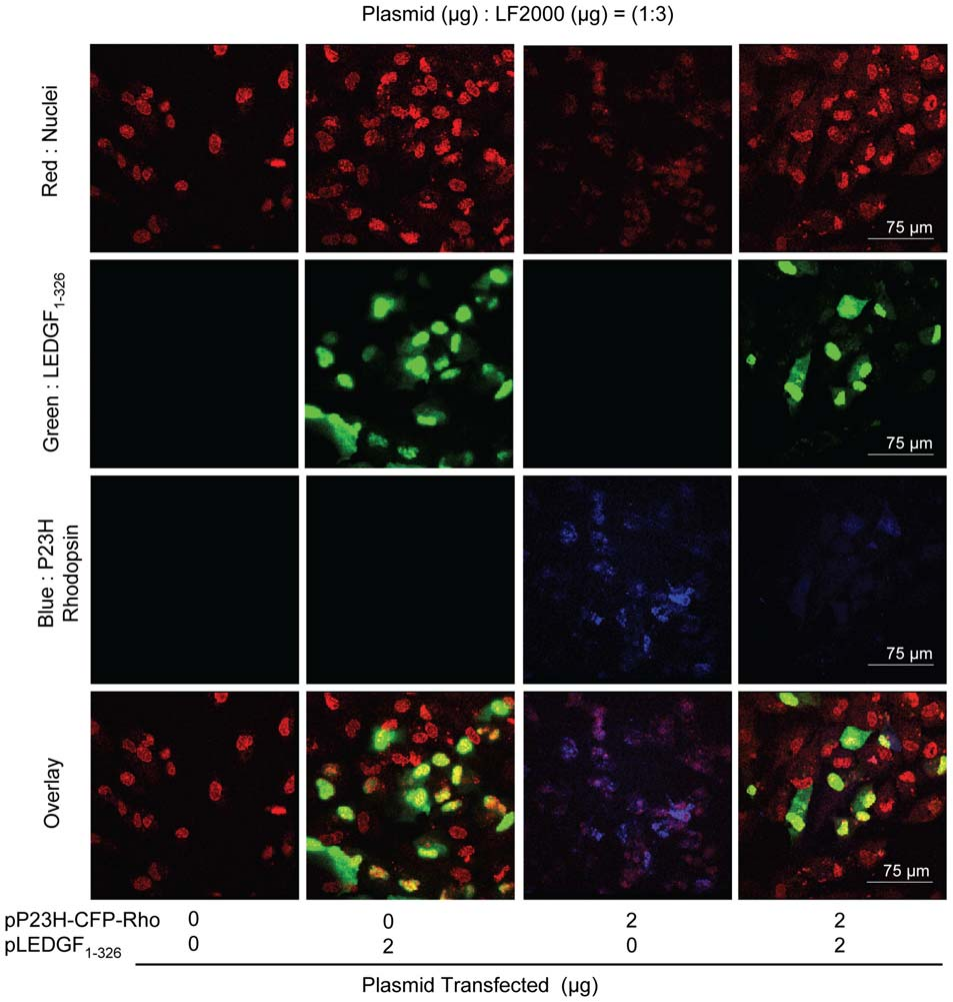
LEDGF_1-326_ reduces P23H rhodopsin mediate nuclear damage. Cells were fixed with 4% buffered formalin, permeabilized with 0.2% Triton X-100 and treated with RNase. The nucleus was then stained with TO-PRO-3 iodide. Confocal images were taken using the excitation/emission wavelengths of 408–450/35, 488–515/30, and 637–605/75 nm for CFP, TO-PRO-3 iodide, and GFP, respectively. Images were analyzed using EZ-C1 3.20 FreeViewer software. Data presents representative images from one of the three independent studies.

To further investigate the role of LEDGF_1-326_ in reducing aggregation, confocal microscopy was performed on cotransfected ARPE-19 cultures (Figure 5A). Nuclei were stained red as previously described, LEDGF_1-326_ expression was visualized by green fluorescence emitted by tagged GFP protein with LEDGF_1-326_, and P23H rhodopsin was visualized as blue. The LF2000 transfected control group (Figure 5, Column 1) indicated clear red nuclei staining and the nuclei exhibited well formed oval structures. The group transfected with pLEDGF_1-326_ (Figure 5, Column 2) showed clear green fluorescence colocalized with the red labeled nuclei, indicating the presence of LEDGF_1-326_ primarily in the nucleus. The group transfected with pP23H-CFP-Rho (Figure 5, Column 3) showed blue fluorescence and once again indicated disrupted nuclear material as before (Figure 4A). However, in the presence of cotransfected pLEDGF_1-326_, disrupted nuclear material (broken, non-uniform red pattern) was decreased and the intact nuclear structure was restored (Figure 5, Column 4). Interestingly, while P23H rhodopsin expression was clearly visible near the perinuclear region of the cell in the absence of LEDGF_1-326_ (Figure 5, Row 3), it was diffused and far less visible in the presence of LEDGF_1-326_ (Figure 5, Column 4).

**Figure 6:**
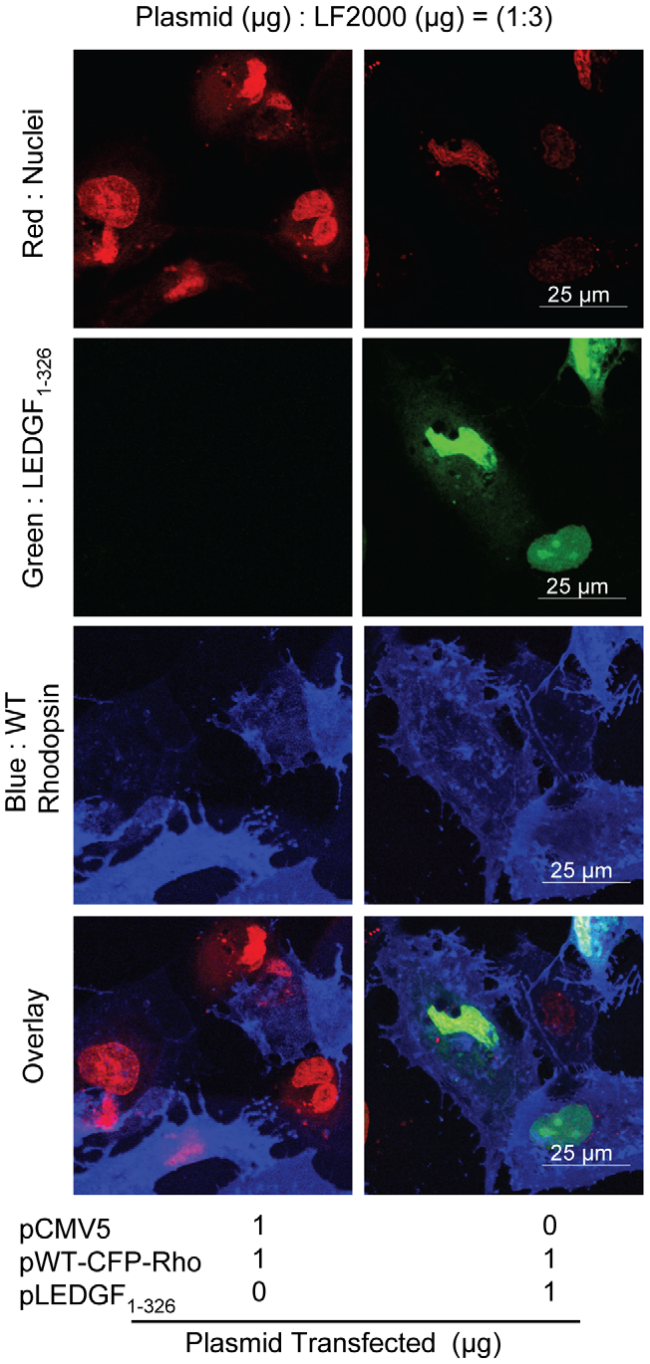
Diffuse fluorescent signal of WT rhodopsin in RPE cells. Cells were fixed with 4% buffered formalin, permeabilized with 0.2% Triton X-100 and treated with RNase. The nucleus was then stained with TO-PRO-3 iodide. WT rhodopsin visualization was done using Zeiss LSM 510 NLO laser scanning confocal microscope with 63x optical zoom. The excitation-emission wavelengths used for CFP, GFP, and TO-PRO-3 iodide were 800 nm (2-photon excitation)-435/485, 488–505 (long pass), 633–650/710 nm, respectively. Images were analyzed using Zen 2000 light edition. Data presents representative images from one of the three independent studies.

WT rhodopsin expression was similarly visualized similarly (Figure 6). Unlike P23H rhodopsin, WT rhodopsin was expressed evenly in the cytoplasm. The plasma membrane was clearly marked by its expression leading us to believe that WT rhodopsin migrated to the plasma membrane after its expression. No disrupted nuclear material was evident due to the expression of WT rhodopsin.

### LEDGF_1-326_ reduces rhodopsin aggregates

**Figure 7:**
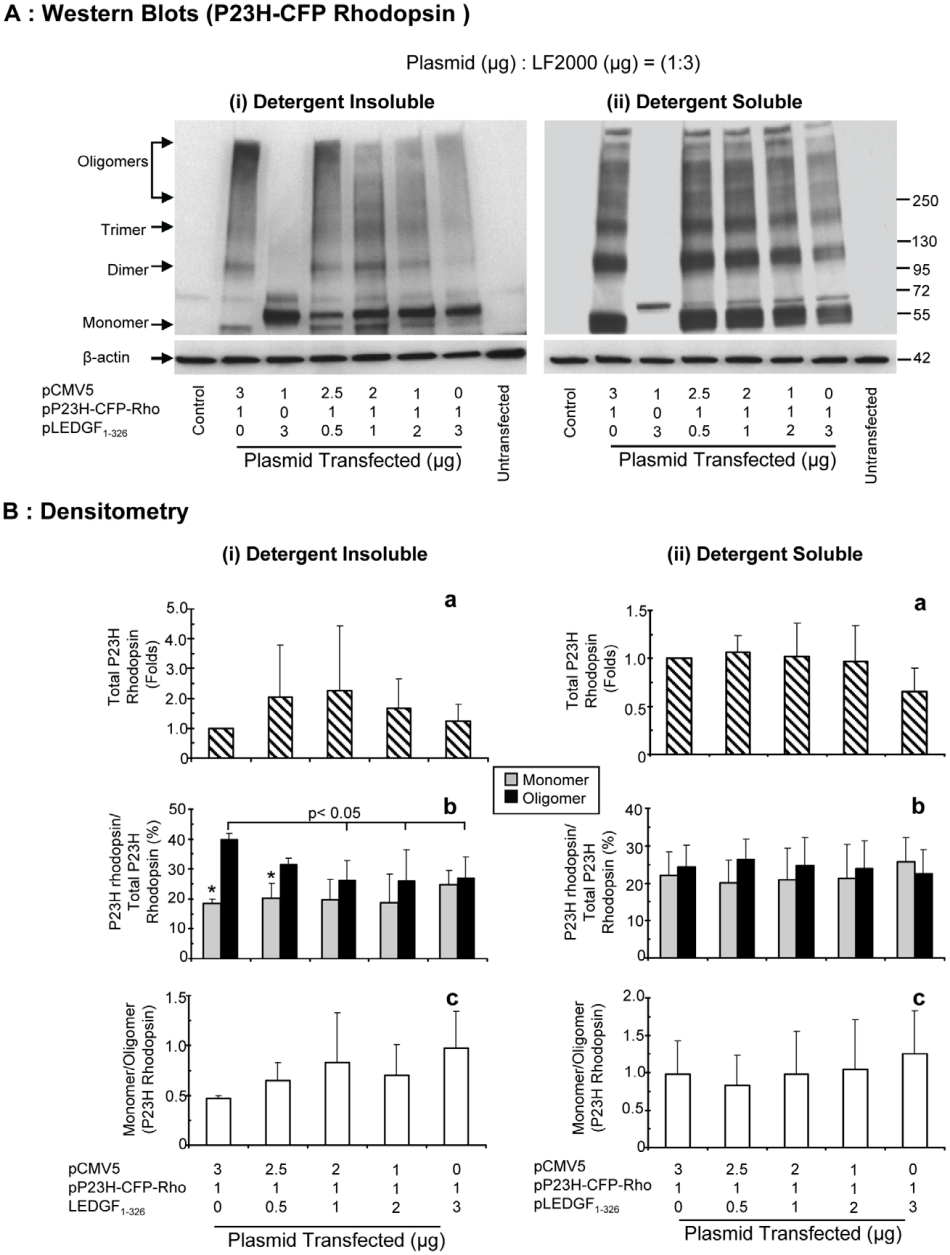
LEDGF_1-326_ decreases the oligomers of P23H rhodopsin in RPE cells in a dose-dependent manner. **A**) For western blotting cells were lysed using 1% Triton X-100 lysis buffer and the detergent soluble fraction (ii) was collected after centrifugation. Thereafter, the detergent insoluble pellet was sonicated using a probe sonicator in 1% SDS buffer, the supernatant was collected by centrifugation and described as detergent insoluble fraction (i). SDS-PAGE was run for both fractions on 4–20% gradient gel. The protein was then transferred onto nitrocellulose membrane and then immunoblotted using rabbit polyclonal anti-CFP antibody and goat polyclonal anti-rabbit. β-actin was probed after stripping the membrane and then immunoblotting with mouse monoclonal anti-actin antibody and sheep polyclonal anti-mouse antibody. The protein was detected using enhanced chemiluminescence film detection method. Representative blot from one of the four independent studies has been shown. **B**) For densitometry analysis equal regions were selected from each lane corresponding to each fraction (monomer, dimer, trimer, and oligomer). Total rhodopsin mean density was calculated by adding all the fractions and then mean density was normalized to β-actin. Data is expressed as mean ± S.D. for N = 4. *, (p<0.05), indicates significant difference from oligomer in the same group.

Aggregation of P23H rhodopsin was monitored by western blotting in the absence and presence of LEDGF_1-326_. Figure 7A represents blots from one of four independent experiments. The band at 50-55 kDa represented the monomeric form of P23H rhodopsin, whereas the dimers and trimers were represented by smears at about 100–120 kDa and 180–200 kDa, respectively. The oligomers represented all higher molecular weight (≥200 kDa) species of P23H rhodopsin.

A high molecular weight smear (representing oligomers) of P23H rhodopsin was seen within the detergent insoluble fraction in all groups transfected with pP23H-CFP-Rho (Figure 7A (i), Lane 2, 4–7). This dark smear gradually diminished as the cells were cotransfected with increasing doses of pLEDGF_1-326_, while keeping pP23H-CFP-Rho constant. At a transfection ratio of 1∶1 of pP23H-CFP-Rho to pLEDGF _1-326_, there was slight increase in total P23H rhodopsin. Interestingly we also observed a decrease in the oligomers and an increase in the monomers at this ratio. However, at transfection ratio of 1∶2 and 1∶3, total P23H rhodopsin was seen to decrease with increasing levels of pLEDGF_1-326_ for both detergent insoluble and soluble portion.

For P23H rhodopsin, the percentage of oligomers in the detergent insoluble fraction decreased from 39±2 to 27±9% in a dose proportionate manner in the presence of LEDGF_1-326_ (Figure 7B(i(b))) (p<0.05). The percentage of monomers increased from 18±1 to 25±4%, but did not attain statistical significance. The P23H rhodopsin oligomer percentage in the detergent insoluble fraction was significantly (p<0.05) higher than the monomer percentage in controls (Figure 7B(i(b)), Group1); but with increasing dose of LEDGF_1-326_, this difference diminished. The monomer to oligomer ratio increased with increasing dose of LEDGF_1-326_ (Figure 7B(i(c))). In the detergent insoluble fraction (Figure 7B), it was apparent that the total P23H rhodopsin abundance increased when the transfection ratio of pP23H-CFP-Rho and pLEDGF_1-326_ was 1∶1, but decreased in the presence of higher levels of pLEDGF_1-326_ (Figure 7B (i(a)). However, these changes were not statistically significant. For the detergent soluble fraction, similar, but less evident trends were seen (Figure 7B (ii)).

**Figure 8:**
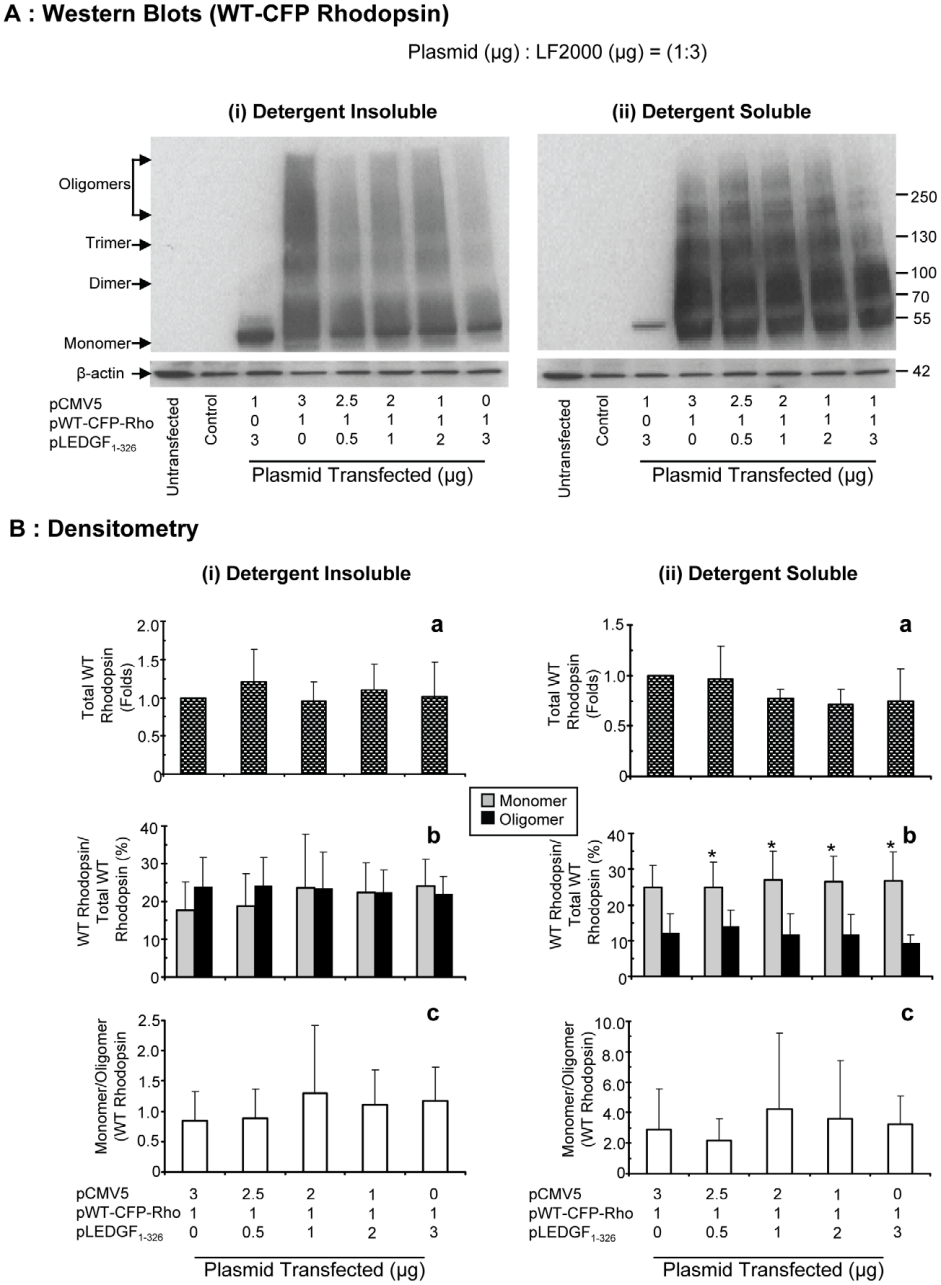
LEDGF_1-326_ decreases the oligomers of WT rhodopsin in RPE cells in a dose-dependent manner. **A**) For western blotting cells were lysed using 1% Triton X-100 lysis buffer and the detergent soluble fraction (ii) was collected after centrifugation. Thereafter, the detergent insoluble pellet was sonicated using a probe sonicator in 1% SDS buffer, the supernatant was collected by centrifugation as detergent insoluble fraction (i). SDS-PAGE was run for both fractions on 4–20% gradient gel. The protein was then transferred onto nitrocellulose membrane and immunoblotted using rabbit polyclonal anti-CFP antibody and goat polyclonal anti-rabbit. β-actin was probed after stripping the membrane and then immunoblotting with mouse monoclonal anti-actin antibody and sheep polyclonal anti-mouse antibody. The protein was detected using enhanced chemiluminescence film detection method. Representative blot from one of the three independent studies has been shown. **B**) For densitometry analysis equal regions were selected from each lane corresponding to each fraction (monomer, dimer, trimer, and oligomer). Total rhodopsin mean density was calculated by adding all the fractions and then mean density was normalized to β-actin. Data is expressed as mean ± S.D. for N = 4. *,( p<0.05), indicates significant difference from oligomer in the same group.

To further investigate whether LEDGF_1-326_ had a similar effect on WT rhodopsin, western blot studies were repeated with pWT-CFP-Rho (wild type rhodopsin tagged with cyan fluorescent protein). Figure 8A represents blots from one of four independent studies. The detergent insoluble (Figure 8A (i)) and detergent soluble fraction (Figure 8A (ii)) indicated a ladder of WT rhodopsin.

For WT rhodopsin in the detergent soluble fraction, the percentage of monomer was higher than the oligomer and this difference increased due to a decline in oligomer percentage and an increase in monomer percentage and became statistically significant (p<0.05) with increasing dose of LEDGF_1-326_ (Figure 8B(ii(b))).There was a decrease in the WT rhodopsin oligomer percentage from 12±5 to 9±2% and an increase in the monomer percentage from 24±6 to 28±7% in presence of LEDGF_1-326_ at the highest dose. Further, the monomer to oligomer ratio in the detergent soluble fraction increased with increasing dose of LEDGF_1-326_ (Figure 8B(ii(c))). In the detergent insoluble fraction, although not statistically significant, the WT rhodopsin oligomer percentage was slightly higher than the monomer percentage in control (Group1) and this difference decreased with increasing dose of LEDGF_1-326_.due to an increase in the monomer percentage. Specifically, there was a decrease in the WT rhodopsin oligomer percentage from 23±7 to 21±4% and an increase in the monomer percentage from 17±7 to 24±7% in the presence of LEDGF_1-326_ (Figure 8B(i(b)). The monomer to oligomer ratio increased with increasing dose of LEDGF_1-326_ (Figure 8B(i(c))). Although statistically not significant, densitometry analysis indicated that, similar to P23H rhodopsin, there was a decrease in the total WT rhodopsin in both detergent insoluble as well as in soluble fraction (Figure 8B(i(a)), and (ii(a))), especially at the highest dose of LEDGF_1-326_. Compared to P23H rhodopsin, the oligomer percentage of WT rhodopsin in each individual group was less (compare Figure 7B(i(b))) and 8B(i(b)))).

Apart from rhodopsin smears, another band was seen at about 65–70 kDa in all blots (Figure 7, and 8). Since this band was well separated from the rhodopsin smears and was visualized only in those groups transfected/cotransfected with pLEDGF_1-326_, it was assumed to be representing LEDGF_1-326_, possibly due to cross reactivity of anti-CFP antibody with GFP tag of LEDGF_1-326_.

### LEDGF_1-326_ has no effect on transcription of P23H rhodopsin

**Figure 9:**
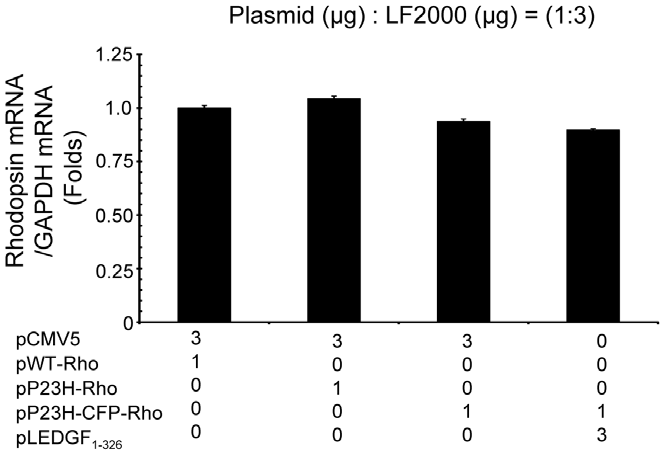
LEDGF_1-326_ does not alter the transcription level of P23H rhodopsin. mRNA was isolated from transfected ARPE-19 cells using the RNeasy kit. To remove contaminating genomic DNA, 10 µg RNA from each sample was treated with DNase using the Turbo DNA free kit. First strand synthesis was done using the high capacity RNA to DNA. PCR was performed to amplify the DNA on an ABI 7500 PCR machine. The threshold thermal cycle was used to calculate the mRNA level of rhodopsin and was normalized to GAPDH mRNA level. Data is expressed as mean ± S.D. for N = 3. Data was considered significant at p<0.01 compared with pWT-Rho transfected group.

To determine if LEDGF_1-326_ had an effect on P23H rhodopsin transcription, we examined the levels of P23H rhodopsin mRNA (Figure 9). Rhodopsin mRNA was measured by qRT-PCR (quantitative real time polymerase chain reaction). No significant difference in the mRNA levels was found between the group transfected with pWT-Rho, pP23H-Rho, and pP23H-CFP-Rho. Further, the mRNA level of P23H-CFP rhodopsin was seen to have no significant change in the absence and presence of LEDGF_1-326_, indicating that LEDGF_1-326_ has no effect on the transcription of P23H-CFP rhodopsin. Thus, the LEDGF_1-326_ mediated decrease in total P23H-CFP rhodopsin abundance is not due to decreased P23H transcription.

## Discussion

The principal findings of this study are: 1) P23H rhodopsin and WT rhodopsin form aggregates in RPE cells 2) LEDGF_1-326_ decreases both P23H and WT rhodopsin aggregates; 3) P23H rhodopsin disrupts nuclei and decreases the viability and proliferation of RPE cells; and 4) LEDGF_1-326_ decreases the cellular damage caused by P23H rhodopsin. Thus, LEDGF_1-326_ might be a suitable therapeutic agent for reducing rhodopsin aggregates and preventing cellular degeneration in diseases like retinitis pigmentosa.

Others have shown that P23H rhodopsin mediated aggregation is not limited to photoreceptors, but rather is a fundamental property of the mutant protein [5]. Our data is consistent with this observation. P23H rhodopsin, when expressed in RPE cells, formed insoluble aggregates and decreased cell viability. P23H rhodopsin likely induced cell death via aggregation mediated stress. RPE cells play an important role in the development and maintenance of photoreceptor cells. They are closely associated with photoreceptor outer segments and phagocytose the constantly shed segments throughout the life of the organism. Additionally, they play an active role in photoreceptor metabolism, taking up waste products and supplying essential metabolic intermediates. Given that RPE take up large quantities of shed outer segments, they are almost certainly exposed to the P23H rhodopsin mutant.

LEDGF_1-326_ partially rescued RPE cultures from P23H rhodopsin mediated cell death as measured by cell viability (Figure 3). LEDGF_1-326_ also appeared to rescue cells from P23H rhodopsin mediated damage as measured by confocal microscopy (Figure 5). In multiple independent experiments, P23H rhodopsin expression resulted in a distinct reduction in the number of nuclei and disruption of nuclear shape and content, indicating nuclear damage (Figure 4, and 5). Cotransfection of pLEDGF_1-326_ appeared to rescue cells from this damage (Figure 5). Compared to pP23H-CFP-Rho transfected group, the group cotransfected with both plasmids indicated a higher number of distinct, normal nuclei and more viable cells. Further, P23H rhodopsin was localized near the perinuclear region, consistent with protein aggregation, as reported by others [5]. Conversely, we observed a significant diffused fluorescence of P23H rhodopsin when the cells were cotransfected with pLEDGF_1-326_. WT rhodopsin, on the other hand, showed diffuse fluorescence pattern in the absence and presence of pLEDGF_1-326_ (Figure 6).

Our western blot analysis indicated that although both P23H and WT rhodopsin aggregated in ARPE-19 cells, the percentage of WT rhodopsin oligomers were less as compared to P23H rhodopsin in both detergent insoluble and soluble fractions (Figure 7 and 8). Further, LEDGF_1-326_ can reduce the aggregates of rhodopsin in RPE cells. As the level of pLEDGF_1-326_ cotransfection was increased, there was a dose-dependent decrease in the oligomers of P23H as well as WT rhodopsin. At low doses, LEDGF_1-326_ reduced P23H and WT rhodopsin oligomers and increased their monomers, without affecting the total rhodopsin. However, at the higher doses of pLEDGF_1-326_ there was also a decrease in the total rhodopsin protein indicating possibly degradation of rhodopsin. This indicates that LEDGF_1-326_ function is complex, apart from deaggregation, it might be assisting the aggregated rhodopsin to degrade. Although LEDGF_1-326_ was present in detergent insoluble fraction, it was not present in oligomeric forms, indicating it is not aggregation prone. Since LEDGF_1-326_ was found primarily in the nucleus (Figure 5), it might be tightly bound to DNA and the initial mild lysis condition (1% Triton-x 100) may not have been sufficient to fully extract LEDGF_1-326_ into the soluble fraction. Thus, LEDGF_1-326_, a protein that may not aggregate by itself, reduces the intracellular aggregates of rhodopsin.

Since our western blots were performed with CFP tagged rhodopsin, there may be a concern that CFP might show aggregation behavior. However, although CFP was tagged to both P23H rhodopsin as well as to WT rhodopsin, the aggregation behavior and cellular damage was more prominent with P23H rhodopsin (Figures 7 and 8). Further, both P23H rhodopsin and P23H rhodopsin tagged with CFP resulted in similar loss in cell viability (Figure 2B). Hence, it appears that CFP itself may have minimal effects, if any, on the observed aggregation behavior of rhodopsin proteins and cytotoxicity of P23H rhodopsin. Further, it was previously demonstrated that CFP fusion at the C-terminal of rhodopsin has no discernable effect on its folding or intracellular distribution [24].

Given that LEDGF has transcriptional activity, it is theoretically possible that coexpression of LEDGF_1-326_ may alter the expression of rhodopsin in our co-transfection experiments. Since the qPCR study (Figure 9) showed no change in the mRNA level of P23H rhodopsin in the presence and absence of LEDGF_1-326_, it appears that LEDGF_1-326_ does not play a role in the transcription of P23H rhodopsin. However, whether LEDGF_1-326_ is acting directly on P23H rhodopsin or exerting its effect via the upregulation of various stress related proteins is unknown at this time.

A previous study conducted using LEDGF protein showed that there was no rescue of b-waves in the electroretinogram (ERG) of P23H transgenic rat model with rapidly degenerating photoreceptors (Line 1), indicating that LEDGF cannot rescue P23H mediated photoreceptor damage in the rat model, when dosed intravitreally on day 10 at a dose of 1 µg [25]. However, the same study showed that LEDGF can rescue photoreceptors in light-damaged and RCS rats. Another study employing AAV vectors capable of expressing LEDGF also showed photoreceptor rescue in RCS rats but not P23H transgenic (Line 1) rat model [26]. While the in vivo studies in P23H rats would seem to contradict our findings, it is possible that once the aggregates of P23H rhodopsin are formed to a certain extent, LEDGF cannot prevent further damage. Dosing at an earlier stage than the previous study and at a higher level might be necessary in order to obtain significant benefits with LEDGF or its derivatives in animal models. Thus, LEDGF_1-326_ is of potential value in treating retinal degenerations associated with P23H rhodopsin.

We have found that the deletion of the HSE from LEDGF has not abrogated its ability to promote cell survival or protein deaggregation, indicating that the HSE domain is not essential for this function. The effect of this deletion on the ability of LEDGF to activate gene expression has not been directly assessed. Deletion studies in which somewhat larger sections of the C-terminus were removed resulted in significant decreases in the transcriptional activity of the construct [18]. Additionally, LEDGF constructs consisting only of N-terminal sequence retained much of their ability to drive the transcription of a reporter gene, indicating that the N-terminus of LEDGF provides domains important for transcription. These data would suggest that LEDGF_1-326_ may be transcriptionally impaired. However, this has not yet been explicitly tested. Further investigations are warranted in this context. Since LEDGF_1-326_ reduced rhodopsin aggregates in RPE cells, we speculate that this contributes at least in part to its ability to reduce nuclear damage and cell death in RPE cells.

RPE degeneration contributes to vision loss in human RP. Our data demonstrates that P23H can be toxic to RPE, suggesting that the uptake of toxic photoreceptor components may play an important role in RP pathogenesis. The degree to which phagocytosed P23H rhodopsin contributes to RPE degeneration will be dependent on the ability of the RPE to process phagocytosed material; an important and underappreciated issue requiring further investigation.

## Materials and Methods

Plasmid pP23H-CFP-Rho was a gift from Dr. Ron R. Kopito, (University of Stanford, Stanford, CA) and pCMV5 was a gift from Dr. David W Russell (University of Texas Southwestern Medical Center, Dallas, Texas). Rabbit polyclonal anti-cyan fluorescent protein (anti-CFP) antibody, goat polyclonal horse peroxidase linked anti-rabbit antibody and CFP protein was purchased from BioVision (Mountain View, CA). ARPE-19 cells were obtained from ATCC (Manassas, VA). DMEM/F12 cell culture medium, fetal bovine serum, LF2000 (Lipofectamine® 2000), and nucleic acid staining dye TO-PRO-3 iodide was obtained from Invitrogen (Carlsbad, CA). All other chemicals, unless specified otherwise, were purchased from Sigma- Aldrich (St. Louis, MO).

### Plasmid construction

pP23H-Rho was cloned from the pP23H-CFP-Rho by cutting out CFP using Ecor1 and Not1 restriction enzymes. The cloned plasmid was ligated and then transformed in E. coli DH5α. The plasmid construct was confirmed for molecular weight by gel electrophoresis and for gene sequence by sequencing.

For point mutation of pP23H-CFP to pRho-CFP and pP23H-Rho to pWT-Rho, the primers used were 5′GGGTGTGGTACGCAGCCCCTTCGAGTACCCACAG3′ and 5′CGTTGGGTACTCGAAGGGGCTGCGTACCACACCC 3′. The mutation was done using the Quick Change kit (Stratagene, La Jolla, CA) as per manufacturer’s protocol. The mutation was confirmed by sequencing the gene.

### Cell transfection

ARPE-19 cells were cultured in DMEM/F12 (1∶1) medium containing 10% (v/v) fetal bovine serum(FBS), 2% (v/v) L-glutamine (200 mM), and 1% (v/v) penicillin-streptomycin (10,000 units/ml of penicillin G sodium mixed with 10,000 µg/ml of streptomycin sulphate) in a cell incubator maintained at 37°C and 5% carbon dioxide as per ATCC protocol. For transient transfection, about 10^5^ cells were plated in 12-well plate and incubated. After 24 h, the medium was aspirated out and the cells were transfected/cotransfected with pP23H-CFP-Rho/pLEDGF_1-326_ (this plasmid contained GFP tag at the N-terminus of LEDGF_1-326_)/pCMV5 (empty vector) using LF2000 in serum free DMEM/F12 medium. Plasmids of different ratios (e.g., increasing pLEDGF_1-326_, with decreasing pCMV5 plasmid, with a constant total plasmid level) were used a) to keep the lipofectamine level in various groups constant and b) to control plasmid transfection efficiency, since an increase in plasmid level can saturate the transfection process. Cells transfected with LF2000 alone (control) were also used. After 8 h of incubation, the transfection medium was removed and the cells were further incubated for 24 h in normal serum containing medium. Thereafter, cells were treated as per individual experiments. All the transfection reagents including cells and plasmids were up scaled to five times for studies done in 60 mm plates according to LF2000 transfection protocol. All figures are labeled to represent the dose exposed as the level used per 10,000 cells. Similar transfection was done for WT rhodopsin.

### Phase contrast microscopy

For phase contrast microscopy, after transfection, cells were kept on ice and live cells were imaged using an inverted light microscope (Nikon Eclipse TE300). Hoffman Modulation Contrast images were taken using 10x optical lens. The images were captured using Image pro® software (Nikon).

### Cell viability assay

After transfection, cells were trypsinized using 0.25% trypsin-EDTA and collected in Eppendorf tubes. They were then centrifuged at 1000 g for 5 min to form a pellet. The supernatant was discarded and the pellet was resuspended in PBS. To this 0.4% of trypan blue was added to stain the dead cells. Unstained viable cells were thereafter counted using Bright-Line haemocytometer (Hausser Scientific, Horsham PA). The percentage viability of cells in individual group was calculated with respect to the LF2000 group.

### Cell proliferation assay

The proliferation assay of ARPE-19 cells was done using bromodeoxyuridine (BrDU) assay kit (Calbiochem, San Diego, CA) as per manufacturer’s protocol. BrDU, a thymidine analogue, gets incorporated into newly synthesized DNA strands as the cells proliferate. Thus, a reduction in percentage of BrDU incorporation is indicative of reduction in percentage of cells proliferating. The number of cells and the transfection reagents were scaled down ten times, as compared to 12-well plate, for transfection in 96-well plate. BrDU label (20 µl) was added to each well along with the serum containing medium after 8 h of transfection. Two types of negative controls were maintained: blank (BrDU label but no cells) and background (untransfected cells with no BrDU label). The cells were further incubated for 24 h and thereafter fixed, permeabilized, and the DNA was denatured. The cells were incubated with mouse anti-BrDU antibody for 1 hour. After washing the unbound antibody, goat anti-mouse antibody conjugated with horseradish peroxidase was added and incubated for 30 min and then tetra-methylbenzidine (TMB), a chromogenic substrate was added and the absorbance of color developed was measured at dual wavelengths of 450 nm and 540 nm using a microplate reader. The absorbance at 450 nm minus the absorbance at 540 nm indicated the proliferation. The percentage proliferation of individual group was then calculated with respect to LF2000 group.

### Confocal microscopy

For confocal microscopy, ARPE-19 cells were grown on cover slips. After transfection, the cells were fixed with 4% buffered formalin for 20 min, and permeabilized with 0.2% Triton X-100 for 10 min. They were then treated with RNase (100 µg/ml) for 20 minutes to prevent staining of RNAs with TO-PRO-3 iodide. The nucleus (nuclear DNA) was then stained with TO-PRO-3 iodide (1 µM) for 15 min. All treatments were done at 37°C unless specified and were followed by three washes with PBS. The cover slip was mounted on a glass slide using Supermount® (Biogenex, San Ramon, CA) mounting media to prevent rapid loss of fluorescence. The slides were allowed to dry for 20 min at room temperature and fluorescence was visualized using confocal microscope (Nikon Eclipse C1) at 60x optical zoom. The excitation-emission wavelengths used for CFP, GFP, and TO-PRO-3 iodide were 408–450/35, 488–515/30, and 637–605/75 nm, respectively. Images were captured using Nikon EZ-C1 software version 3.40. For nuclei count, images from three independent studies were taken and the number of distinct nuclei per frame in each image was counted and the mean number of distinct nuclei per group was plotted against the level of pP23H-CFP-Rho transfected.

For WT-CFP rhodopsin visualization, Zeiss LSM 510 NLO laser scanning confocal microscope was used at 63x optical zoom. The excitation-emission wavelengths used for CFP, GFP, and TO-PRO-3 iodide were 800 nm (2-photon excitation)-435/485, 488–505 (long pass), 633–650/710 nm, respectively. Images were analyzed using Zen 2000 light edition software (Carl Zeiss, Thornwood, NY).

### Western blotting

For western blotting, cells were cultured in 60 mm dishes. The number of cells and the transfection reagents were scaled up five times. After transfection, the cells were lysed for 30 min using 200 µl of lysis buffer (5 mM EDTA, 1% Triton X-100, protease inhibitor (Complete Mini, Roche Diagnostic, IN) in PBS) under ice-cold conditions. The lysed cells were collected and centrifuged at 13000 g for 10 min. The supernatant was collected (named as detergent soluble fraction) and the pellet was further redissolved into 50 µl of PBS containing 1% SDS at room temperature for 10 min. Then 150 µl of lysis buffer was added and samples were sonicated for 20 sec using a probe sonicator (Mesonix 3000) set at 3 Watts. The cells were recentrifuged and the supernatant (named as detergent insoluble fraction) was collected. Protein estimation of both the detergent soluble and the insoluble fraction was done using BCA protein assay reagent (Pierce, Rockford, IL). For gel electrophoresis the samples were mixed with 4x loading dye, however, boiling was avoided to prevent heat induced aggregation of rhodopsin. For detergent soluble fraction 40 µg and for detergent insoluble fraction 30 µg of protein was loaded for each experimental group. CFP protein was loaded for positive control. Samples were run on 4–20% gradient SDS-PAGE gel (Bio-Rad, Hercules, CA) and then transferred to nitrocellulose membrane. The membrane was then immunoblotted using rabbit polyclonal anti-CFP antibody (1∶1000) as primary antibody and anti-rabbit antibody (1∶10000) as secondary antibody. Protein bands were visualized using enhanced chemiluminecence ECLTM detection kit (GE Healthcare, Piscataway, NJ), and high performance chemiluminescence films (GE Healthcare, Piscataway, NJ). Thereafter, the membrane was stripped with stripping buffer containing 2% SDS, and 0.1 M beta-mercaptoethanol at 50°C for 15 min and reprobed for β-actin protein using mouse monoclonal anti-actin antibody as primary antibody (1∶1000) and sheep anti-mouse antibody (1∶10000) as secondary antibody. Similarly, WT rhodopsin immunoblotting was also done.

Densitometry analysis was done using Quantity One 1-D analysis software from Bio-Rad. Equal regions from individual lanes of blots were selected and the mean intensity of each region was measured and normalized for β-actin. Thereafter, the mean intensity was subtracted from the corresponding region of the untransfected group to reduce the background noise. The mean intensity of corresponding lane and protein fraction was then plotted for rhodopsin transfected groups.

### Real-Time quantitative PCR

ARPE-19 cells were transfected as above and thereafter RNA was isolated from cultured cells using the RNeasy kit (Qiagen, Valencia CA) according to the manufacturer’s protocol. To remove contaminating genomic DNA, 10 µg RNA from each sample was treated with DNase using the Turbo DNA free kit (Ambion, Austin, TX) as per user’s manual to degrade any DNA present in the sample. Thereafter, first strand of DNA was synthesized from the mRNA using the high capacity RNA to DNA kit (Applied Biosystems, Inc., Carlsbad, CA). Then, PCR was performed on an ABI 7500 PCR machine to amplify the DNA. The number of thermal cycle required to reach threshold was obtained. Rhodopsin mRNA level was calculated from this threshold cycle. Similarly GAPDH mRNA (mRNA commonly present in all cells) level was detected and rhodopsin mRNA level was normalized to GAPDH mRNA to represent the mRNA level of rhodopsin present in each group.

### Statistical analysis

All statistical analysis was done using SPSS, ver. 19; (SPSS, Chicago, IL). The data is represented as the mean ± SD, Independent samples student’s t-test was done for statistical comparisons between two experimental groups. To compare between multiple experimental groups one-way ANOVA followed by the Tukey’s post hoc analysis was used. The results were considered statistically significant at p<0.05.
